# Transoral robotic surgery in head and neck district: a retrospective study on 67 patients treated in a single center

**DOI:** 10.1186/s13027-020-00306-7

**Published:** 2020-06-15

**Authors:** Fraco Ionna, Agostino Guida, Luigi Califano, Gaetano Motta, Giovanni Salzano, Ettore Pavone, Corrado Aversa, Francesco Longo, Salvatore Villano, Ludovica Marcella Ponzo, Pierluigi Franco, Simona Losito, Franco Maria Buonaguro, Maria Lina Tornesello, Maria Grazia Maglione

**Affiliations:** 1 Maxillofacial & ENT surgery Unit, Istituto Nazionale Tumori-IRCCS “Fondazione G. Pascale”, via M. Semmola, Naples, Italy; 2 Department of Neurosciences, Reproductive and Odontostomatological Sciences, Director and Chair, Maxillofacial Surgery unit, University of Naples “Federico II”, Naples, Italy; 3 Department of Neuroscience, Reproductive and Odontostomatologic Sciences, ENT Unit, University “Federico II”, Naples, Italy; 4 Departement of Pathology, Istituto Nazionale Tumori-IRCCS “Fondazione G. Pascale”, Naples, Italy; 5 Molecular Biology and Viral Oncology Unit, Istituto Nazionale Tumori-IRCCS “Fondazione G. Pascale”, Naples, Italy

**Keywords:** Trans oral robotic surgery, Head and neck surgery, Oropharynx, de-intensified treatment

## Abstract

**Background:**

The anatomical complexity of the oropharynx and the difficulty in reaching its distal portion have always conditioned the surgical accessibility.

Robotic surgery represents an excellent alternative in the treatment of cervico-facial oncological diseases.

**Methods:**

This series comprises all patients managed for head and neck cancer by Trans Oral Robotic Surgery TORS.

The staging assessment, including neck ultrasound and total body PET/CT scan, was performed in each patient according to the TNM classification.

All charts were recorded with the following data: name and surname, age, gender, date of surgery intra or post-operative hemorragia, tumor site, histology, TNM stage, robot set-up time, tumor resection time, whether or not tracheotomy was performed, whether or not neck dissection was performed, insertion of a nasogastric tube or gastrostomy, time to resumption of oral feeding, surgical margins, mean length of hospital stay, adjuvant treatment and follow-up.

**Results:**

From February 2013 to February 2018, TORS was performed in 67 consecutive patients affected by head and neck tumours.

We divided, our sample, in 3 subsites: supraglottic larynx, parapharyngeal space and oropharynx.

Pathology reports confimed malignancy in 44 cases: 8 cases lymphomas, 36 cases of Squamous cell carcinoma (SCC), 5 cases of benign salivary glands tumors and 18 miscellaneous cases. Neck dissection was performed in 12 cases.

Tracheotomy was perfomed in 3/67 cases for respiratory failures. A nasogastric tube was inserted at the end of the surgical procedure in 21 patients. The mean length of hospital stay was 10 days .

Major complications included post-operative bleeding in 3 patients, 1 exitus for massive bleeding 20 days post-surgery and 1 respiratory failure treated with tracheotomy and monitoring in the Intensive Care Unit (ICU) for 3 days.

**Conclusions:**

Robotic surgery has been considered a valid alternative to traditional open treatment in many specializations with the advantages of an endoscopic procedure, with the same oncological and functional results and with fewer complications. The advantages of this type of surgical technique have been discussed, it is mandatory to focus on the indications and contraindications.

## Introduction

The anatomical complexity of the oropharynx and the difficulty in reaching its distal portion have always conditioned the surgical accessibility, limiting the traditional transoral resection possibilities to the upper oropharynx (soft palate and tonsillar regions).

For decades the surgical gold standard was the trans-mandibular approach, but this was burdened by important morbidities and high complication rates.

Robotic assisted surgery was first described in 1976 when NASA began to study a method to assist astronauts in orbit by using a technology that allowed telepresence surgery [1].

The fusion between telecommunications and robotic technology allows the surgeon to operate remotely from the main operating field with the same accuracy, using the robot as his hands and visualizing the operating field more accurately with the aid of 3D technology.

After the first laparoscopic splenectomy performed in 1997 [2], robotic surgery has gained popularity in many specialties such as general surgery, urology, cardiac surgery, gynecology and neurosurgery [3].

Trans oral robotic surgery (TORS) was introduced for the first time by Weinstein et al., who in 2005 described a case of supraglottic laryngectomy on a canine model [4]; MacLeod and Melder instead reported again in 2005 the excision of an epiglottic vallecular cyst in a patient, with a surgical set-up of 75 min including an effective operative time of 30 min [5].

Since that time robotic surgery has been considered a valid alternative to traditional open treatment in many specializations.

TORS is indicated as a valid therapeutic / surgical alternative in numerous studies on cadavers, animals and for the treatment of various oesophageal tumors.

The use of TORS and the Da Vinci Robot was described in literature for the first time in 2006 and then later in 2007 by a group of University of Pennsylvania surgeons who demonstrated the safety and flexibility of this procedure for transoral resection of hypopharynx tumors [6, 7]. Subsequently, in December 2009 the US Food Drug Administration approved TORS for the resection of benign and malignant tumors of the head neck district [8].

It has therefore been established in the literature that robotic surgery represents an excellent alternative to open, endoscopic and microscopic surgery in the treatment of cervico-facial oncological diseases as it improves the vision of the first operator, is easier to use and reduces operating times.

The goal of our study is to report our TORS experience. To the best of our knowledge this is the first report that highlights the results of using TORS in such a large and heterogeneous sample of patients treated in a single center.

## Materials and methods

This series comprises all patients managed for head and neck cancer by TORS between February 2013 and February 2018 by the MaxilloFacial and ENT surgery department of Istituto Nazionale Tumori IRCCS Pascale, Naples, Italy.

The staging assessment, including neck ultrasound and total body PET/CT scan, was performed in each patient according to the TNM classification (8edt).

Each case was discussed at a multidisciplinary consultation meeting during which it was decided to perform TORS.

Unilateral or bilateral neck dissection of group I to V nodes, according to tumour site, was performed during the same operating time, when indicated.

Patients underwent general anesthesia via nasotracheal intubation. Transoral exposure was obtained with a Feyh–Kastenbauer (FK) retractor and three arms were used: a central endoscopic arm with a 0° integrated three-dimensional camera; a right robotic arm with a 5-mm monopolar cautery with a spatula tip; and a left robotic arm with a 5-mm DeBakey forceps. The surgeon was seated at the console and the assistant was seated at the patient’s head to monitor the operative site, retract tissues, and facilitate dissection, to evacuate smoke released by the monopolar electrosurgery, and to perform suction in the case of intraoperative bleeding.

Adjuvant therapy was discussed at the multidisciplinary consultation meetings, based on the pTNM classification.

All charts were recorded with the following data: name and surname, age, gender, date of surgery, intra or post-operative hemorragia, tumor site, histology, pTNM stage, robot set-up time, tumor resection time, whether or not tracheotomy was performed, whether or not neck dissection was performed, insertion of a nasogastric tube or gastrostomy, time to resumption of oral feeding, surgical margins, mean length of hospital stay, adjuvant treatment and follow-up.

## Results

**Fig. 1:**
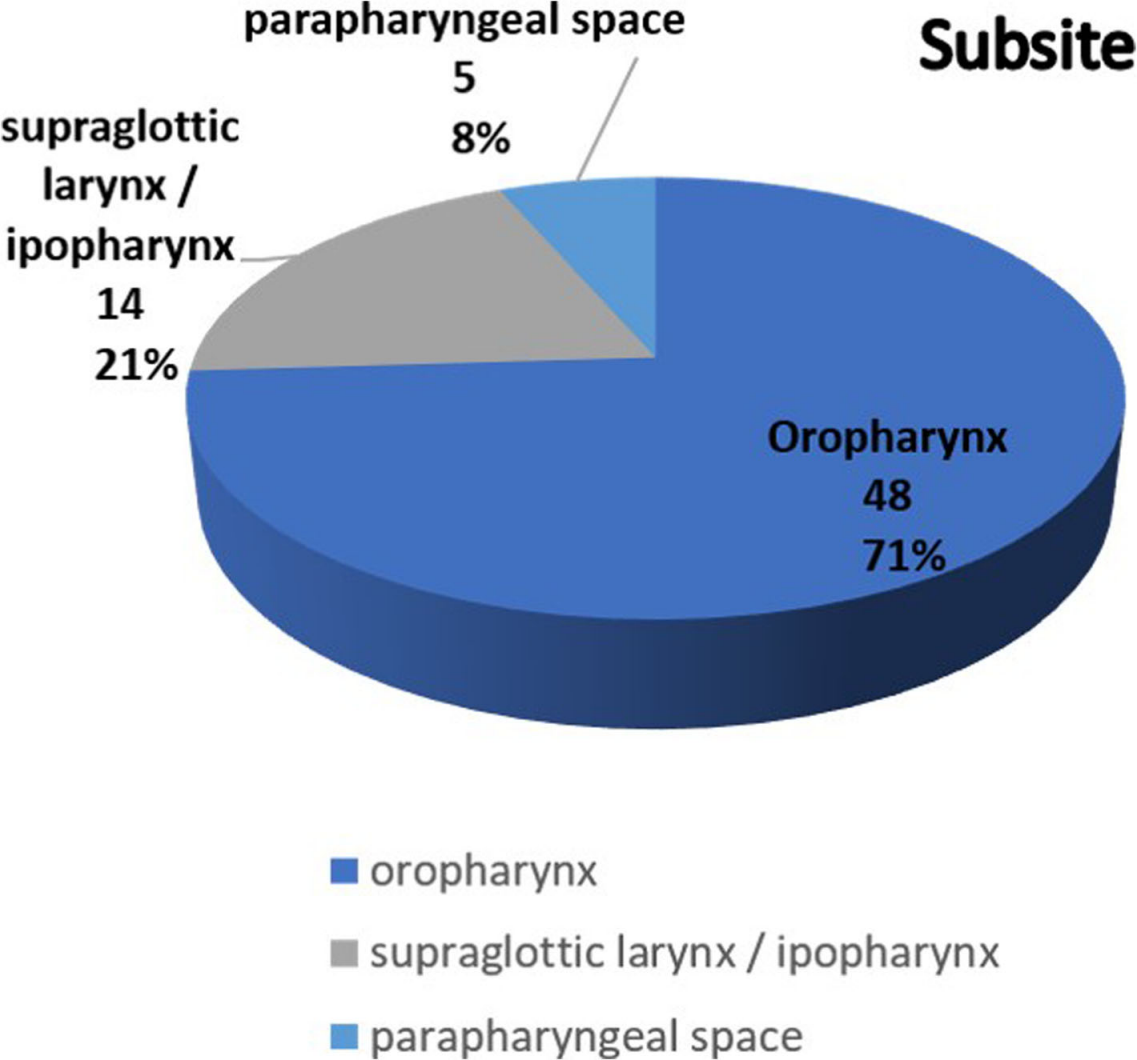
Subsites: Anatomical distribution of primary tumour

**Fig. 2:**
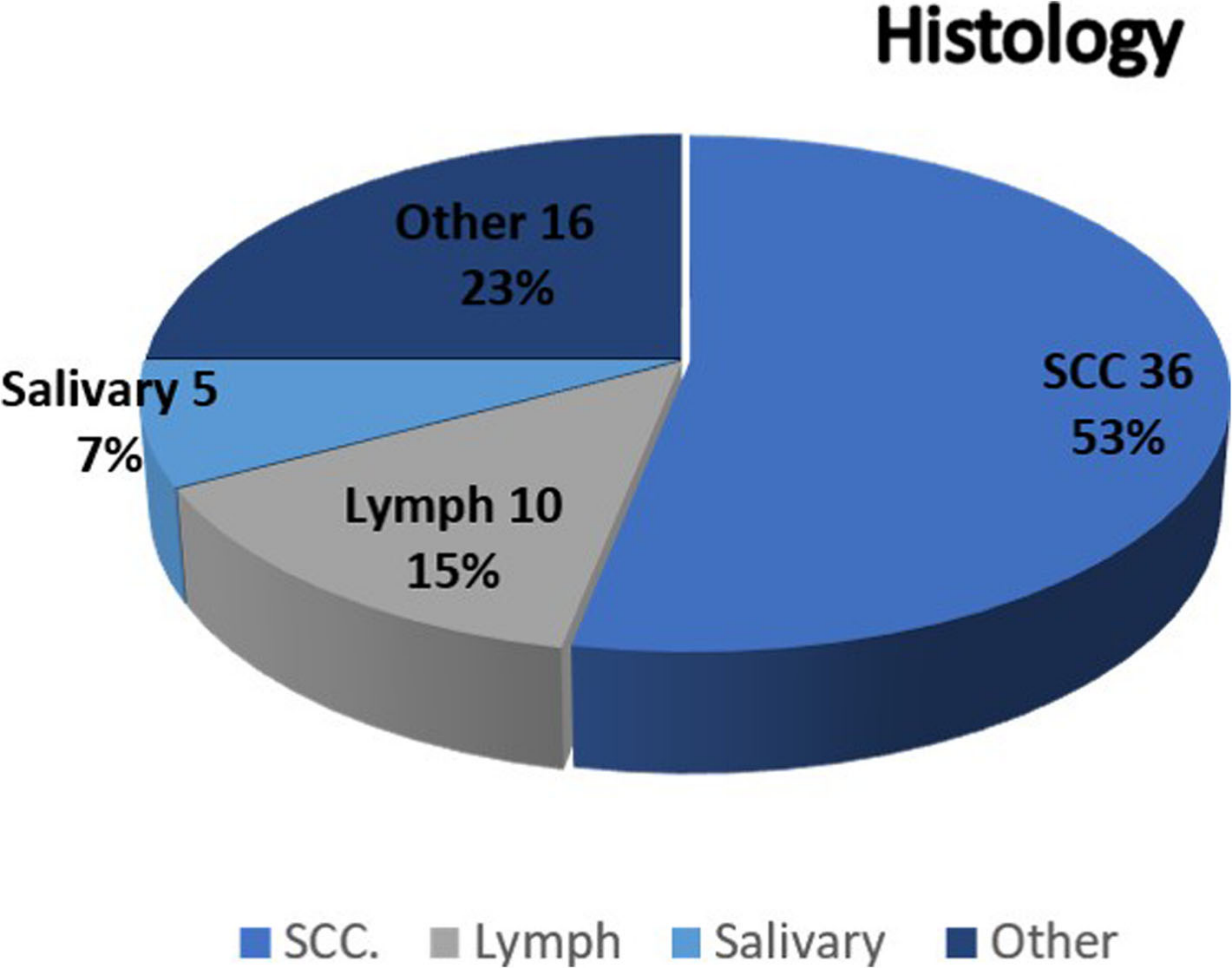
Histology: Distribuition of histological diagnosis

From February 2013 to February 2018, TORS was performed in 67 consecutive patients affected by head and neck tumors at the MaxilloFacial and ENT surgery department of Istituto Nazionale Tumori IRCCS Pascale, Naples, Italy. The mean age of the patients was 56.2 years (range 18–85), with a male/female ratio of 45–22. We divided, our sample, in 3 subsites (Fig. 1): supraglottic larynx 18 cases (27%), parapharyngeal space 5 cases (7%) and oropharynx 44 cases (66%) (Fig.1). The final pathology (Fig. 2) report confimed malignancy in 44 cases: 8cases (12%) lymphomas, 36 cases of Squamous cell carcinoma (SCC) (54%), 5 cases of benign salivary glands tumors (7%) and 18 miscellaneous cases (27%) (Fig.2).

In lymphomas cases we had a limph-node fnab indicative for immunoproliferative process but was mandatory histological characterization, moreover the oropharynx localization gave in all cases severe dysphagia and in 2 cases oropharynx localization were the only recurrence site.

Regarding the 36 cases of SCC clear resection margins (> 5 mm) were obtained in 12 cases, positive margins were found in 14 cases and close resection margins (< 0.5 mm) were identified in 10 cases. The HPV status for 11 patients of the 36 SCC cases (30%) was positive.

Unilateral or bilateral dissection (2 cases) was performed in 12 cases (18%) during initial surgery with a diagnosis of SCC > T1,

Tracheotomy was perfomed in 3/67 (4%) cases for respiratory failures. A nasogastric tube was inserted at the end of the surgical procedure in 21 patients (31%). The other patients did not require a nasogastric tube and resumed normal oral feeding on D2. The mean length of hospital stay was 10 days (3–40).

Major complications included post-operative bleeding in 3 patients, 1 exitus for massive bleeding 20 days post-surgery and 1 respiratory failure treated with tracheotomy and monitoring in the Intensive Care Unit (ICU) for 3 days .

The mean robot set-up and surgical resection times were 22.4 [± 11] and 42.3[± 21] minutes, respective.

One case was converted to a transoral blunt instrument and finger approach to complete the caudal dissection of the mass(a parapharingeal mass).

Frozen section examination of the operative specimens was performed in all patients to study the surgical margins.

2 patients received adjuvant radiotherapy (RT), 19 patients were treated with chemoradiotherapy (CT + RT), 1 patient was treated with chemotherapy (CT) this group underwent a TORS procedure in advanced local diseases for reduction and debulking purpose; finally and 1 patient was treated with a combination of RT+ surgery on N and CT.

Post-operative pain management 7 days after surgery was mainly performed with NSAIDs or paracetamol (75%) or elastomeric pump infusion (25%) (ketorolac + tramadol + ranitidine).

The follow-up was performed over a period ranging from 2 months for the patients operated more recently up to 5 years with the following results:

regarding the patients with a malignant histology, 36 patients were free of disease (no evidence of disease NED), 3 patients had died of disease (DOD), 6 patients are currently receiving combined radio / chemo treatment (alive with disease AWD) and 1 patient at the last control showed the presence of a second neoplasm in another site (breast carcinoma), with a disease free survival (DFS) of 78.2% and an overall survival (OS) of 93.4%.

## Discussion

In the past, most surgery for oral and pharyngeal carcinomas (OPC) was performed through transfacial or transmandibular incisions [8, 9]. Although these approaches sometimes give a better visualization of the neoplastic lesion, these surgical techniques have been replaced in the treatment of cT1-T3 squamous cell tumors of the oro/ hypopharynx, first by TLM mini-invasive laser surgery and then by TORS. These new surgical approaches may provide improved functional outcomes with minimal surgical morbidity [9].

TORS, in fact, associated in N+ cases with neck dissection, allows the ablation of the tumor mass with a non-invasive approach that guarantees a 3D view of the surgical field and therefore the possibility of dominating the neoplasm with “safe” margins.

The trans-oral approach was first introduced by Huet in 1955 [10]. Steiner [11] was the first to introduce the concept of trans-oral laser microsurgery for the treatment of benign and malignant neoplasms of the oropharynx associated with the use of the rigid 3D laryngoscope (TLM).

From the second half of the 2000s, TORS displaced the endoscopic approach, increasing the surgical possibilities, thanks to the overcoming of the technical limits of TLM. This technique offers many advantages: no visible external scars, the 360° motion of the robotic system, three-dimensional high definition visualization, and reduction of hand tremors allow good manipulation of the tissues, improved visualized surgical field, and a shorter postoperative length of hospital stay [9, 10].

Another fundamental concept is “de-intensified treatment”, that represents the reduction of chemotherapeutic drug doses and of the radiotherapy field of action.

There are two ongoing ECOG 3311 studies (Phase II Randomized Trial of Transoral Surgical Resection followed by Low-dose or Standard-dose IMRT in Resectable p16 + Locally Advanced Oropharynx Cancer) and PATHOS (phase II / III trial of risk-stratified, reduced intensity adjuvant treatment in patients undergoing transoral surgery for Human papillomavirus (HPV) positive oropharyngeal cancer) using information about unfavorable prognostic factors, obtained from histological examination, to stratify patients according to risk and thus propose intensifying therapies where possible [12–25].

In 2009, the US Food and Drug Administration approved the use of the da Vinci Surgical System for transoral otolaryngology. Previously, the treatment of OPC had been reserved for a combined radio and chemotherapeutic approach with good results in terms of survival, but linked to acute and late toxicity. Similiar complications were connected with trans-mandibular approaches that guaranteed a good resection with important deficits for the patient and irreversible changes in the quality of life (QOL).

These considerations are the basis of the concept of de-intensified treatment that allows us to take advantage of TORS and therefore a minimally invasive surgery with positive results in terms of survival [25, 26].

Optimizing the quality of life of these patients, reducing the toxicity of the radiation dose to be administered, without compromising the possibilities of treatment, has become imperative [25, 26].

Today it is therefore possible to reduce the radio-chemotherapy treatments in “intensity”, with a consequent decrease in morbidity.

In support of this thesis, TORS is an emerging treatment option in order to modulate the adjuvant therapy [13–17] avoiding invasive and disabling surgical approaches.

The TORS data are encouraging, the parameters studied being the surgical feasibility ie the analysis of the set-up time and operational time and oncological and functional outcomes (gastrostomy dependence, functional assessment of swallowing, need for tracheotomy and.

QoL) [27–30].

Once the advantages of this type of surgical technique have been discussed, it is mandatory to focus on the indications and contraindications.

Mouth opening must be evaluated to ensure a correct positioning of the robotic arms and exposure of the working space. Trismus (mouth opening of less than 1.5 cm), macroglossia, and mandibular/maxillary defects may represent a major contraindication to performing TORS. Furthermore, much is still debated regarding the predictive markers of contraindication to TORS, and even preoperative cephalometric analysis has been proposed. MRI is reliable in ruling out carotid encasement and bone erosion [30].

Nevertheless, tissue adherences may complicate dissection, and if these are located in the lateral/posterior part of the mass, towards the external carotid, a change of approach to transoral blunt instrument and finger dissection may be required. As suggested by other authors, the intraoperative decision to switch to a transoral blunt dissection may be necessary − or even advisable - in order to guarantee patient safety (minimize the risk of major haemorrhaging from the carotid artery) and tumor capsule integrity in the absence of particular predictive radiographic signs [30, 31]. Although in literature a percentage of 4.5% of cases that require a conversion to an open approach is described [32], in our series one case (a parapharyngeal mass) was converted to a transoral blunt instrument and finger approach to complete the caudal dissection of the mass, due to severe lateral adherences limiting the safety and efficacy of the dissection.

We analyzed sufficient mouth opening in all patients allowing adequate exposure of the tumour;

accurate diagnostic workup for the selection of cases is necessary to the indication of TORS.

Furthermore, patients must have transoral access to the oropharynx. T1,T2 oropharyngeal cancers are amenable to resection, above all if they arise from the lateral tongue base, the tonsillar fossa, the lateral pharyngeal wall and the glossopharyngeal sulcus [9]. Particular attention should be paid to patients who have undergone previous radiotherapy treatments, who have rheumatologic diseases or patients who need antithrombotic or anticoagulant therapy, because in these patients TORS involves a greater risk of postoperative bleeding as with this technique the wounds heal secondarily [9].The reported bleeding rates after TORS have ranged considerably from 1.5 to 11.5% in recent studies [31]. These rates are derived from studies that are highly variable in cohort size and the criteria used to define post-TORS [33]. Our case bleeding rates are 4.4% (only 3 cases out of 67).

Regarding the literature, Weinstein et al. reported 100% locoregional control for T1–3 tonsillar carcinomas in 27 patients [7]. De Almeida JR et al. in 2015 published a report of 410 patients treated in 11 centers with TORS demonstrating a 2-year locoregional control rate of 91.8% and an overall survival rate of 91.0% [34]. Patients with tonsillar tumors had better locoregional control compared with patients with tumors of the facial arch, and the oncological outcomes seemed to be independent of any association with HPV. Moreover, in 2015 Holsinger and Ferris [9] reported a review of the fundamentals of transoral endoscopic head and neck surgery, with robotics and laser technology, and discussed ongoing clinical trials for patients with OPC focusing on the indications, the contraindications, the functional results and the protocol of therapeutic de-escalation.

Still debated in the literature is the relationship between HPV and TORS and there are still no clear indications about the possible differences in the use of TORS in patients with positive or negative HPV. Ang et al. [14] highlighted the significant impact of HPV status with 720 patients with T2–4 N1–3 OPC. Patients with HPV-positive tumors had an overall survival rate of 82.4% compared to 57.1% for patients with HPV-negative (*P* < 001). Other smaller studies do not suggest any prognostic impact of the status of HPV for T1–2 patients with resectable OPC if treated with an initial TORS approach [35].

In addition, doubts have been raised regarding the high rate of adjuvant radiotherapy in TORS patients but a study by Weinsten et al. [36] reported 97% local control with minimal follow-up and 90% regional control for 30 selected TORS patients who did not receive any postoperative adjuvant therapy.TORS may also be selectively used for patients with recurrent disease after primary radiation therapy with or without chemotherapy [9]. A recent publication by White et al. [37] compared the role of TORS versus standard open surgery in patients who underwent surgical salvage treatment. Patients treated with TORS were found to have a significantly lower incidence of tracheostomy use (*P* < 001) and feeding tube use (P < 001) and shorter overall hospital stays. The 2-year recurrence-free survival rate was significantly higher in the TORS group than in the open approach group (74 and 43%, respectively; *P* < 01). In our series only 5 cases out of 67 (7.46%) underwent surgical salvage treatment for local advanced diseases previously treated with CHT and RT.

Regarding the comparison between TORS and open head and neck surgery, a large review of the National Hospital Data Survey database between 1995 and 1997 including 3932 patients in non-robotic head and neck procedures [38] looked at patients having operations of the pharynx for malignancy. The major complication rate was 9.3% and a mortality rate of 2.33% was reported.

In our study we found a major complication rate of 5.97% and a mortality rate of 4.4%.

Hammoudi et al. in 2015 [38] compared TORS procedures with conventional surgery in the treatment of SCC of the upper aerodigestive tract in 2 groups of 26 patients. The report showed significantly fewer tracheotomies in the TORS group, 4 vs 20 respectively (*p* < .001). The mean durations of feeding by nasogastric tube and hospitalization were shorter for the TORS group (*p* = .001). There was no significant difference in disease-free survival at 3 years (*p* = .76). The mean treatment cost was $7124 lower for the TORS group (*p* = .03). In our group we performed only 3 tracheotomies in 67 procedures (4%).

From our experience it has emerged that the robot allows us to operate in patients with benign tumors, diagnostic lymphomas, miscellaneous and T1-T2-T3 OPC in narrow and anatomically complex spaces, while for the more advanced stages radiant treatment is preferable.

In lymphomas cases we had a limph-node fnab indicative for immunoproliferative process but was mandatory histological characterization, moreover the oropharynx localization gave severe dysphagia and in 2 cases oropharynx localization was the only recurrence site.

Regarding patients with a malignant histology, 36 patients were free of disease (no evidence of disease NED), 3 patients died of disease (DOD), 6 patients are currently receiving combined radio / chemo treatment (alive with disease AWD) and 1 patient at the last control showed the presence of a second neoplasm in another site (K breast), with a disease free survival (DFS) rate of 78.2% and an overall survival (OS) rate of 93.4% with a mean follow up of 30 months.

The most frequent sequela reported by our patients was postoperative pain controlled with paracetamol or with the use of NSAIDs 7 days after surgery in 60% of patients.

TORS allows us to perform transversally the same interventions that can be carried out with the classic open procedure, but with the advantages of an endoscopic procedure, with the same oncological and functional results and with fewer complications.

It is possible to avoid invasive procedures such as mandibulotomy, reduce the range of radiotherapy (de-intensified treatment), reduce the need to perform a tracheotomy, avoid the onset of problems related to a possible iatrogenic malocclusion, reduce intra and post-operative bleedings, reduce the possibility of infections due to the less extensive surgical site, favor a faster functional recovery by decreasing the number of patients undergoing enteral nutrition in the post-operative period, decrease the presence of visible scarring and shorten hospitalization times.

From a literature review it has emerged that the problems of the management of patients undergoing major cancer surgery can be addressed with the choice of TORS so reducing average hospitalization times [39].

With robotic surgery the same results can be achieved but with a lower morbidity [40].

The current trend is to consider robotics for T1-T2 the indication for this surgery are currently being extended to glottic tumours and larger lesions such as some T3 -T4 tumors [41, 42].

We performed a TORS procedure in local advanced diseases for disobstructive and cytoreductive aims and to obtain better and broader histological material for histopathological characterization;

this explains the number of positive margins.

In conclusions TORS represents a good tool for staging and treating neoplasms of head and neck cancer [43–46], the development of these minimally invasive surgical techniques offers a significant opportunity to impact positively on patient quality of life and post treatment function with a satisfactory oncologic control but further studies on larger series will be needed to confirm the results obtained and to well define the indications and the contraindications of TORS.

## Data Availability

The datasets used and/or analysed during the current study are available from the corresponding author upon reasonable request.
